# Acute Pancreatitis as the Initial Presentation of Systematic Lupus Erythematosus

**DOI:** 10.1155/2014/571493

**Published:** 2014-08-14

**Authors:** Yi Jia, Arleen Ortiz, Richard Mccallum, Hasan Salameh, Pedro Serrato

**Affiliations:** Department of Internal Medicine, Paul L. Foster School of Medicine, Texas Tech University Health Sciences Center, 4800 Alberta Avenue, El Paso, TX 79905, USA

## Abstract

Systematic lupus erythematosus (SLE) is a multisystem disease, including the gastrointestinal system in about half of SLE patients. As a rare complication of SLE, acute pancreatitis presents as generalized flare-ups in most cases of patients previously diagnosed with SLE. Here we report a rare case of acute pancreatitis as the initial presentation with later diagnosis of SLE.

## 1. Introduction

Systematic lupus erythematosus (SLE) related pancreatitis is an exclusive diagnosis that could be made only after ruling out other possible etiologies. Diagnosis of SLE pancreatitis by itself is usually based on clinical symptoms, laboratory tests, and tomographic findings. The following case describes a patient who developed acute pancreatitis as the initial presentation of systematic lupus erythrematous (SLE).

## 2. Case Report

A 23-year-old female presented with complaints of abdominal pain and vomiting. The onset of constant moderate abdominal pain in right upper quadrant, radiating to the right lower quadrant, was 2 weeks prior to admission following a large meal and was progressively worsening. Associated symptoms consisted of cramps and nonbloody vomiting for 2 days. No other exacerbating factors were identified. Relieving factors included analgesics and supine positioning. Review of systems was positive for leg swelling two weeks prior. There was no contributory past medical history, past surgical history, or social history. Patient denies any drug allergies. Medication included Tylenol for pain.

**Figure 1:**
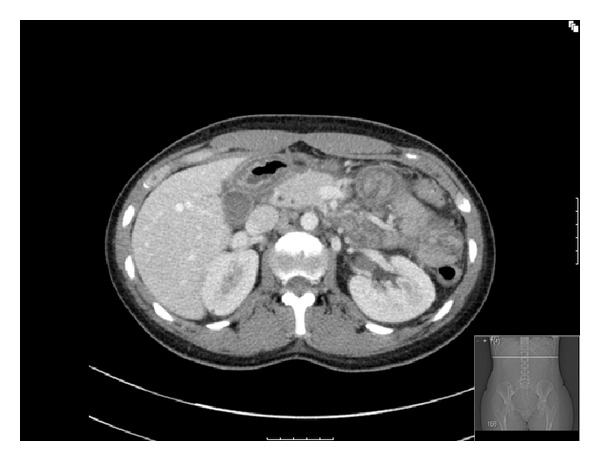
Abdominal computed tomography (CT) scan at admission indicated a thickened edematous bowel loop adjacent to pancreas with mild homogenous edema of pancreas.

Upon physical examination (PE), the patient was afebrile with stable vital signs. Patient's abdomen was moderately distended with right upper and lower quadrant tenderness. Bowel sounds (BS) were absent. No rebound/guarding and no masses or organomegaly were palpable. Other PE findings were unremarkable. Complete blood count and comprehensive metabolic panel were within normal limits. Lipase was 751 units/L. Urinalysis was positive for large blood with only 5–10 RBC per high power field and more than 300 mg protein as per the dip. Other laboratories, including lipid panel, C-reactive protein, and pregnancy test, were all normal. Electrocardiogram, chest X-ray, and abdominal ultrasound were unremarkable. Abdominal computed tomography (CT) scan indicated a thickened edematous bowel loop adjacent to pancreas with mild homogenous edema of pancreas (Figure 1).

**Figure 2:**
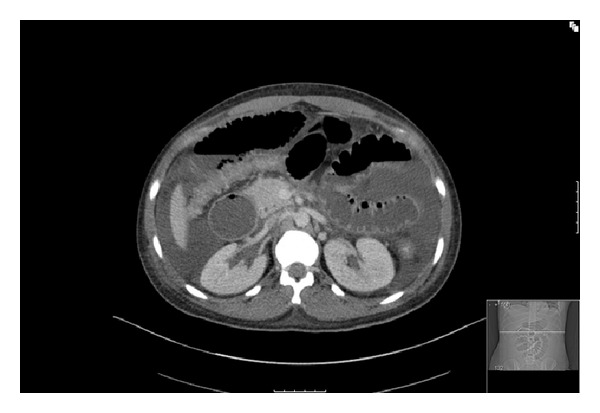
Repeat contrast enhanced CT of the abdomen and pelvis at day 3 after admission revealed pneumatosis involving the distal jejunum, proximal ileum, and diffusely dilated small bowel loops as well as segmented submucosal thickening of the small bowel.

Patient was admitted with a diagnosis of acute pancreatitis and supportive treatment was initiated. During the initial hospital course, the patient's clinical condition deteriorated. The patient developed shortness of breath and abdominal distention and the abdominal pain increased. Patient was found to have bloody stool. Interval PE revealed crackles at lung bases bilaterally. Patient had diffuse abdominal tenderness with absent bowel sound. Repeat contrast enhanced CT of the abdomen and pelvis revealed pneumatosis involving the distal jejunum, proximal ileum, and diffusely dilated small bowel loops as well as segmented submucosal thickening of the small bowel (Figure 2). There was a marked interval increase in volume of ascites and no evidence of focal pancreatic lesions or pseudocysts. Chest CT indicated an isolated segmental partial filling defect in the right middle lobe extending to the subsegmental branch and bilateral pleural effusion with lobar compressive atelectasis. There also was bilateral axillary lymphadenopathy described by the radiologist as likely reactive. Urinalysis of patient again was found to have blood and protein, with a urine sediment revealing dysmorphic RBC and RBC casts. A spot urine protein/creatinine ratio estimated a 24-hour proteinuria to be around 2.5 grams per day. Ranson's score increased from 1 at initial admission to 4 at the third day after admission. With the history of leg swelling and alopecia, further studies were done.

**Figure 3:**
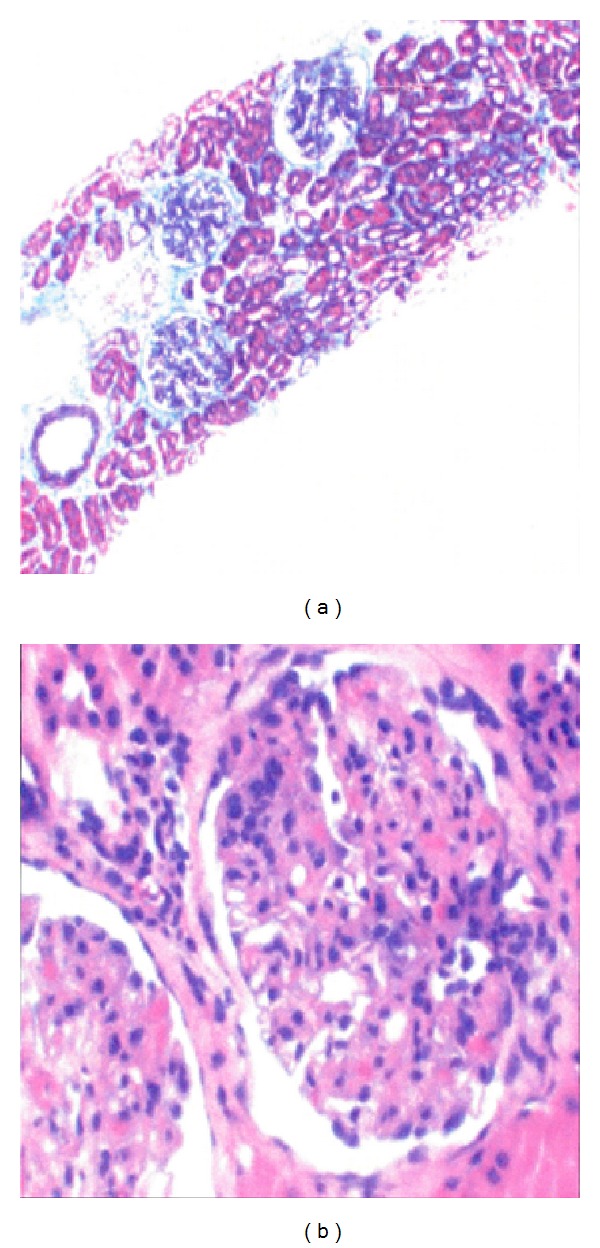
Kidney biopsy of the patient indicated class III lupus nephritis. Less than 50 percent of glomeruli are affected by light microscopy. The glomerulonephritis is almost segmental.

New laboratory test results indicated erythrocyte sedimentation rate 52 mm/h, positive antinuclear antibody (ANA) 1 : 2516, positive anti-Double Stranded (DS) DNA Antibody 1 : 320, rheumatoid factor <10 units/mL, IgG4 150 mg/dL, protein S 29 mg/dL, Von Willebrand factor ristocetin cofactor 372 units/dL, C3 18 mg/dL, C4 < 1.5 mg/dL, lipase 1784 units/L, amylase 302 units/L, and fecal occult blood test positive. Normal Fibrogen level, normal prothrombin time, normal international normalized ratio and partial thromboplastin time, normal hepatitis panel and normal liver enzyme panel. Based on clinical and laboratory criteria, the diagnosis of SLE with possible lupus nephritis and mesenteric vasculitis was made and the patient was treated with methylprednisolone, levofloxacin, metronidazole, bactrim, cyclophosphamide, and mesna. Within days of treatment, patient had clinical improvement and was discharged home with tapering dosage of prednisone. One month after discharge, patient had kidney biopsy (Figure 3), which indicated class III lupus nephritis. On follow-up with nephrology, the patient's renal function was found to be preserved with a urine analysis showing 0–3RBC, no protein, and no casts. Proteinuria improved to less than 300 mg in 24 hours.

## 3. Discussion

Systematic lupus erythematosus (SLE) related pancreatitis is an exclusive diagnosis that could be made only after ruling out other possible etiologies. Gallstones induced obstruction of the pancreatic duct and alcoholic induced pancreatic toxicity are the two most common causes of pancreatitis in the United States [1]. SLE is a multisystem disease, including the gastrointestinal system in about 50% of all SLE patients [2, 3]. As a rare complication of SLE, the frequency of pancreatitis in SLE patients is only about 0.2%–8.2% and presents as generalized flare-ups in most cases of patients previously diagnosed with SLE [4, 5]. Patients with acute pancreatitis had higher systemic lupus erythematosus disease activity index (SLEDAI) scores and higher mortality, compared to those SLE patients without pancreatitis [5, 6]. Only 99 cases of SLE pancreatitis had been documented in the literature and only 10 cases had reported pancreatitis as an initial presentation of SLE [1, 6–11]. Our presented patient had initial SLEDAI score of 8 points (4 points from hematuria, 2 points from decreased C4, and 2 points from alopecia), which increased to 8 points (added 8 points from psychosis and 4 points from proteinuria) in day 3 after admission without the intervention for SLE.

Pathophysiology of SLE pancreatitis had been debated since the first report. SLE pancreatitis can occur as the generalized flare or during disease quiescence. The main risk factors for SLE related pancreatitis included hypertriglyceridemia, psychosis, recent viral infection, and drug toxicity [4, 5]. The underlying mechanisms of SLE may be associated with complement activation and autoimmune reactions, viral infection, hypotension and microthrombi formation, vasculitis, and intimal thickening [11–13]. Long-standing SLE patients commonly have prolonged pharmacological treatments, such as corticosteroids, diuretics, and azathioprine, all of which can cause acute pancreatitis [12]. The increasing documentations of pancreatitis as an initial manifestation of SLE supported that SLE is the primary etiologic factor of SLE pancreatitis [11].

Diagnosis of SLE pancreatitis by itself is usually based on clinical symptoms, laboratory tests, and tomographic findings. The clinical manifestations of SLE pancreatitis can range from subclinical (an elevation of pancreatic enzymes without clinical symptoms) to acute (severe and/or fulminant) to chronic (self-limiting) disease course. In 30.5% of asymptomatic SLE patients, studies have reported hyperamylasaemia [14, 15]. The incidence of subclinical pancreatitis is much higher than symptomatic pancreatitis [7, 14]. Tomographic findings in acute and severe pancreatitis have a diagnostic accuracy of only 70% to 90% [16]. In our patient, the acute pancreatitis was diagnosed based primarily on the clinical symptoms and the laboratory remarkably elevated lipase and amylase. The positivity for ANA and anti-ds DNA serum titers along with the renal biopsy confirmed lupus nephritis. Although we detected the increased serum IgG4 level, the negative pancreatic tomographic findings did not meet the diagnostic criteria of other possible diagnoses, such as autoimmune pancreatitis.

Steroid treatment for acute pancreatitis in SLE patients is controversial because of steroid-induced toxic effect [12]; however, this concern is regarded as minimal. Because the immunosuppressive effect of steroids can significantly improve prognosis in patients with acute pancreatitis, recent studies recommend the administration of steroids during the acute episode of SLE pancreatitis [17–19]. Recent studies also report that SLE-associated pancreatitis has a much higher mortality rate than in SLE patients without pancreatitis (45% versus 3%), particularly when combined with other complications, such as infections, shock, and renal and respiratory insufficiency [17]. Our patient had improved renal function after the treatment of corticosteroids.
